# Pimozide enhances antigen cross-presentation and promotes cytotoxic CD8+ T cell immunity by stimulating dendritic cells

**DOI:** 10.3389/fimmu.2026.1946712

**Published:** 2026-10-01

**Authors:** Luz María Palacios, Nicolás Daniel Dho, Cintia Liliana Araujo Furlán, Guadalupe López Menichetti, Federico Ruiz Moreno, Laura Guantay, Sofía Daiana Castell, María Esperanza Felici, Gastón Soria, Belkys Maletto, María Inés Crespo, Gabriel Morón

**Affiliations:** 1 Departamento de Bioquímica Clínica, Facultad de Ciencias Químicas, Universidad Nacional de Córdoba, Córdoba, Argentina; 2 Centro de Investigaciones en Bioquímica Clínica e Inmunología (CIBICI), Consejo Nacional de Investigaciones Científicas y Técnicas (CONICET), Córdoba, Argentina

**Keywords:** adjuvant, cross-presentation/priming, cytotoxic T lymphocytes (CTL), dendritic cells, pimozide

## Abstract

The activation of cytotoxic CD8^+^ T cells requires antigen presentation in the context of major histocompatibility complex class I (MHC-I). While endogenous antigens can directly access this pathway, the priming of CD8^+^ T cells against exogenous antigens—such as those derived from tumors or internalized pathogens—depends on dendritic cell (DC)-mediated antigen cross-presentation. Strategies capable of enhancing DC cross-presentation are therefore of considerable interest for the development of next-generation immunotherapies. Here, we demonstrate that pimozide, a clinically approved antipsychotic with reported antitumoral and immunomodulatory properties, acts as a pharmacological enhancer of antigen cross-presentation. We show that pimozide-treated DCs display improved cross-presentation of soluble antigens, leading to stronger CD8^+^ T cell activation and proliferation *in vivo*, enhanced IFN-γ production by antigen-specific CD8^+^ T cells *in vitro*, and augmented cytotoxic T lymphocyte responses *in vivo*. Mechanistically, pimozide reshapes antigen-processing pathways in DCs by increasing reactive oxygen species production and altering endosomal physiology, thereby creating a biochemical environment that favors antigen cross-presentation. Moreover, pimozide treatment reduces STAT5 levels in DCs, and pharmacological inhibition of STAT5 with an independent inhibitor recapitulates the enhancement of cross-presentation and CD8^+^ T cell activation, suggesting STAT5 signaling as a mediator of this effect. Together, these findings reveal an unrecognized immunomodulatory activity of pimozide on DCs and establish a conceptual framework for the pharmacological repositioning of small molecules to reprogram DC function. This work ultimately opens new avenues for amplifying cytotoxic CD8^+^ T cell immunity in the context of next-generation cancer immunotherapies.

## Introduction

The development of robust cytotoxic CD8^+^ T cell responses is a critical goal in the design of immunotherapies against cancer and chronic infections. To elicit CD8^+^ T cell responses, antigenic peptides must be presented in the context of major histocompatibility complex class I (MHC I) molecules. Two main pathways mediate this process. The first is the classical MHC I presentation pathway, in which primarily endogenously synthesized proteins such as self-proteins, and viral proteins produced by infected cells, are processed by the proteasome and subsequently loaded onto MHC I molecules. The second is antigen cross-presentation, performed almost exclusively by dendritic cells (DCs), in which extracellular proteins or those derived from pathogens that do not access the cytosol of DCs are internalized and processed to generate peptides for MHC I loading (1–4). This process is essential for inducing CTL responses against antigens derived from tumors or pathogens that would otherwise fail to be presented in the MHC I context.

DCs are specialized antigen-presenting cells that comprise a heterogeneous population mainly composed of conventional DCs (cDCs) and, to a lesser extent, plasmacytoid DCs (pDCs) (5). cDCs are further subdivided into cDC1 and cDC2, both capable of antigen presentation and naïve T cell priming. cDC1 are particularly specialized in antigen cross-presentation to CD8^+^ T cells, owing to their distinctive endocytic and antigen-processing machinery (6–11). In contrast, cDC2 are primarily associated with the orchestration of CD4^+^ T cell immunity; however, several studies have shown that, under specific inflammatory conditions or in the presence of certain adjuvants, cDC2 can also acquire the ability to cross-present antigens (12–16).

The functional competence of DCs — particularly their capacity to cross-present antigens and prime cytotoxic T cells — is increasingly recognized as a key determinant of immunotherapy efficacy and vaccine-induced protection. Clinical and experimental evidence consistently associates the abundance and activation state of cDC1 with enhanced CD8^+^ T cell responses and improved outcomes in both cancer and infectious disease settings (13, 17–25). However, DCs do not operate as a fixed entity: their cross-presenting capacity is dynamically shaped by metabolic programming, endosomal physiology, and intracellular signaling pathways (3, 13, 26–28), all of which represent potential pharmacological targets. However, the repertoire of small molecules capable of selectively enhancing DC intrinsic functions remains limited. Identifying compounds that can reprogram DC antigen-processing machinery to amplify cytotoxic immunity therefore represents a compelling and broadly applicable therapeutic strategy.

Pimozide is a diphenylbutylpiperidine antipsychotic originally developed for the treatment of neuropsychiatric disorders (29, 30). Beyond its neurological indications, numerous studies have reported anti-tumoral, anti-infectious, and immunomodulatory properties for this compound, highlighting its potential for pharmacological repurposing (31–36). In various cancer models, pimozide interferes with cell proliferation and survival through mechanisms involving STAT5 inhibition, reactive oxygen species (ROS) generation, and metabolic alterations (31, 32, 37–41). Importantly, pimozide displays lysosomotropic properties, enabling its accumulation within acidic vesicles and modulation of their pH and dynamics (31, 32, 34, 42). Because endosomal and lysosomal compartments are central to antigen processing and cross-presentation in DCs, drugs with lysosomotropic behavior have emerged as valuable tools to enhance this process (43–46). Based on this concept, we hypothesized that pimozide could exert similar effects by modulating endosomal physiology in DCs, thereby enhancing their capacity to cross-present antigens and stimulate cytotoxic T cell responses.

In this study, we investigate the cellular and molecular effects of pimozide in DCs, characterizing its impact on antigen translocation, ROS generation, and endosomal dynamics. We demonstrate that pimozide enhances antigen cross-presentation and CD8^+^ T cell activation. Collectively, our findings establish pimozide as a pharmacological enhancer of DC cross-presenting function and support the broader concept of repositioning small molecules as DC-targeted immunomodulators to strengthen antigen-specific cytotoxic immunity.

## Materials and methods

### Mice and cell culture

Female C57BL/6 mice (6–8 weeks old) were used for all experiments. OT-I transgenic mice, expressing a T cell receptor specific for the chicken egg albumin (OVA)-derived peptide SIINFEKL (OVA_257–264_ or S8) presented by H-2K^b^, were kindly provided by Dr. F. A. Goldbaum (Fundación Instituto Leloir, Buenos Aires, Argentina). NLRP3-deficient mice (B6.129S6-Nlrp3^tm1Bhk^/J, strain 021302) were obtained from The Jackson Laboratory. All mice were bred and maintained under specific pathogen-free conditions at the animal facility of our institution. All experimental procedures were approved by the Institutional Committee for Animal Care and Use of the Facultad de Ciencias Químicas, Universidad Nacional de Córdoba (CICUAL-FCQ-UNC).

The CD8^+^ T cell hybridoma B3Z, specific for the H-2K^b^-SIINFEKL complex, was kindly provided by Dr. N. Shastri (University of California, Berkeley, USA) and maintained in complete RPMI medium supplemented with hygromycin B (#10 843 555 001; Roche Diagnostics; Germany) and geneticin (#10131-019; Invitrogen; UK). Complete RPMI medium consists of RPMI-1640 (#31800089, Thermo Fisher Scientific, Waltham, USA) containing 10% heat-inactivated FBS (Natocor^®^; Argentina), 1% L-alanyl-l-glutamine dipeptide (#35050-61; GlutaMAX™-I; Thermo Fisher Scientific), antibiotics (#15140-122;100 IU/mL penicillin, 100 µg/mL streptomycin; Thermo Fisher Scientific) and 5 x 10^–5^ M 2β-mercaptoethanol (Sigma-Aldrich, Merck, St. Louis, USA).

Bone marrow–derived dendritic cells (BMDCs) were generated from C57BL/6 mice by culturing bone marrow precursors for 10 days in complete RPMI medium supplemented with 10% (v/v) SP2.0-conditioned medium containing Flt3L (47). The Flt3L-secreting SP2.0 cell line was kindly provided by Dr. Claude Leclerc (Institut Pasteur, Paris, France) and maintained in complete RPMI medium. For some experimental settings, DCs were cultured with 5 µg/mL CpG-ODN 1826 (a TLR-9 agonist with sequence TCCATGACGTTCCTGACGTT and phosphorothioate backbone, synthesized by Operon Technologies -Qiagen-, CA, USA).

The JAWSII DC cell line, used for the antigen translocation assay, were kindly provided by Dr. Ignacio Cebrián (IHEM-CONICET, Mendoza, Argentina) and maintained in Minimum Essential Medium α (MEMα; #11900024, Thermo Fisher Scientific) supplemented with 20% FBS, GlutaMAX™-I, antibiotics and 2β-mercaptoethanol, as described for complete RPMI medium plus GM-CSF produced by J558 cells. The J558 GMCSF-producing cell line was kindly provided by Dr. S. Amigorena (Institute Curie, Paris, France).

Throughout this manuscript, the term ‘dendritic cells’ (DCs) refers to bone marrow-derived dendritic cells (BMDCs) unless otherwise stated.

### Experimental design and animal welfare considerations

The C57BL/6 mouse model was selected because it is the standard genetic background for well-validated experimental models used in antigen cross-presentation studies, including OT-I transgenic mice and NLRP3-deficient mice. This background enables antigen-specific CD8^+^ T cell responses to OVA-derived peptides to be precisely tracked and quantified using well-established tools.

The experimental unit for *in vivo* experiments was a group of animals receiving the same treatment. Each group consisted of a minimum of 4 mice. Sample sizes were determined based on prior experience with the experimental models used in the laboratory and are consistent with previously published studies using equivalent approaches. No formal *a priori* power calculation was performed; group sizes were kept to the minimum compatible with reliable detection of the expected effects, in accordance with the 3Rs principle of reduction. A sensitivity analysis indicates that, for a one-way ANOVA with three groups and n = 4 animals per group at α = 0.05, the design provides 80% power to detect effect sizes of f ≥ 1.07 (statsmodels v0.15.0, Python), corresponding to differences well within the range of those observed in this study.

Animals were allocated to experimental groups by random assignment at the time of immunization. No formal randomization algorithm was applied. For *in vivo* experiments, each mouse group was housed in a separate cage under identical conditions. Cage location was not systematically rotated. Criteria for excluding animals from the study included body weight loss of 10% or more prior to or throughout the experiment, occurrence of injuries, or overt signs of distress. Based on these criteria, no animals were excluded from the analyses presented in this study, and all mice that completed immunization and tissue harvest were included. Blinding was not applied during data collection or analysis.

Mice received intravenous injection of DCs via the retro-orbital plexus using 30G needles in a maximum volume of 100 µL, with 1–10 × 10^5^ cells/mouse. Prior to injection, mice were anesthetized using a 20% v/v isoflurane-propylene glycol mixture administered in an inhalation chamber (48) and received topical ophthalmic anesthesia with 0.5% proparacaine eye drops (49–51). At the end of the experiment, mice were euthanized via cervical dislocation. Death was confirmed by the absence of heartbeat and spontaneous respiration for at least 5 minutes following euthanasia.

The animal facility complies with the Guide to the Care and Use of Experimental Animals published by the Canadian Council on Animal Care and holds assurance number A5802–01 from the Office of Laboratory Animal Welfare (NIH).

### Flow cytometry

Single-cell suspensions were incubated with anti-CD16/32 (clone 2.4G2, produced in-house) for 15 min at 4 °C to block Fc receptor–mediated nonspecific binding and subsequently stained with fluorochrome-conjugated antibodies for 20 min at 4 °C in polypropilene V-bottom 96-well plates. Antibody clones and fluorochromes are listed in **Supplementary Table 1**. For intracellular cytokine staining, splenocytes were stimulated with 2 µg/mL SIINFEKL peptide (synthesized by the Laboratorio Nacional de Investigación y Servicios en Péptidos y Proteínas, Universidad de Buenos Aires, Argentina) in complete RPMI medium for 5 h at 37 °C in the presence of BD GolgiStop™ (#554724, BD Biosciences, San Jose, USA). Cells were surface-stained, fixed, and permeabilized using the BD Cytofix/Cytoperm™ Plus Kit (#554715, BD Biosciences, San Jose, USA) according to the manufacturer’s instructions, followed by intracellular staining for cytokines. OVA-specific CD8^+^ T cells were identified using PE-conjugated H-2K^b^–SIINFEKL tetramers (NIH Tetramer Core Facility, Emory University, USA). Splenocytes were blocked with anti-CD16/32, incubated with tetramer for 1 h at room temperature, and then surface-stained. Flow cytometry was performed using BD LSRFortessa™ X-20 or FACSCanto™ II (both BD Biosciences), or Attune™ NxT instruments (Thermo Fisher Scientific). Data was analyzed with FlowJo software (version 10, BD Biosciences). Antibodies were purchased from BD Biosciences, eBioscience, Invitrogen, or BioLegend (see **Supplementary Table 1**).

### Cell sorting

In selected experiments, Flt3L-BMDCs were sorted into cDC1, and cDC2 subsets. Single cell suspensions of Flt3L-BMDCs were preincubated with anti-CD16/32 (clone 2.4G2) for 15 min at 4 °C to block nonspecific binding of Abs to Fc receptors and then stained with fluorochrome-labeled antibodies for 20 min at 4 °C. Fluorochromes and clones are specified in **Supplementary Table 1**. DCs subsets were defined as: cDC1s: CD11c^+^ MHCII^high^ CD24^high^ SIRPa^-^, cDC2s: CD11c^+^ MHCII^high^ SIRPa^high^. Sorting strategy is shown in **Supplementary Figure S1c**. Sorted populations consistently showed ≥98% purity.

### B3Z activation assay

Flt3L BMDCs (1.0x10^5^ cells/well) or JAWSII cells (0.5x10^5^ cells/well), both in ps-U-bottom-96-well plates, were incubated with 1 mg/mL chromatographically purified OVA (#LS003054, Worthington) in the presence or absence of 15 µM pimozide (#16222, Cayman Chemical, Ann Arbor, USA) for 3–5 h at 37 °C. When indicated, lactacystin (#70980, Cayman Chemical), 10 mM N-acetylcysteine (NAC, #T0875, TargetMol), or 10 µM diphenyleneiodonium (DPI, #T7191, TargetMol) were added 30 min prior to antigen exposure. Cells were washed and co-cultured overnight with B3Z cells (1x10^5^ cells/well). B3Z activation was quantified by measuring β-galactosidase activity using 0.15 mM chlorophenol red-β-D-galactopyranoside (CPRG, Roche Diagnostics GmbH, Mannheim, Germany), 9 mM MgCl_2_ (Cicarelli Laboratorios) and 0.125% NP40 detergent (IGEPAL CA 630, Sigma-Aldrich) in PBS. Absorbance at 595 nm was measured after 4 h of incubation.

### DCs activation, H-2K^b^-SIINFEKL complex expression and stability on DCs surface

DCs (3x10^6^ cells/well in ps-flat-6-well plates) were incubated with 15 µM pimozide for 12 h at 37 °C and culture supernatants were harvested for cytokine production measurement. Cells were then stained with an antibody cocktail containing classical activation markers to be analyzed by flow cytometry.

DCs (2x10^5^ cells/well in ps-U-bottom-96-well plates) were pulsed with 100 ng/mL SIINFEKL peptide, in the presence or absence of 15 µM pimozide for 15 min at 37 °C. Cells were then extensively washed, stained with an APC-conjugated antibody against H-2K^b^-SIINFEKL complex (clone 25-D1.16), and chased for the indicated times (0, 40, 80, 120, 160 min). At each time point, a PE-conjugated anti-IgG secondary antibody was used to amplify the detection of remaining surface complexes. Subsequently, flow cytometry staining was performed with DC markers and complex expression and stability over time was analyzed within the CD11c^+^ MHCII^+^ H-2K^b^-SIINFEKL^+^ population.

### β-lactamase assay

This assay is modified from Cebrián et al. (52). Briefly, JAWSII (1.5x10^5^ cells/well in ps-U-bottom-96-well plates) were pulsed with purified 20 μg/ml β-lactamase at 37 °C. After 30 min, cells were extensively washed and pimozide stimulus was added for another 2 h at 37 °C. Cells were washed and incubated with 1 μM CCF4 probe (#K1095, Thermo Fisher Scientific) for 1.5 h at RT. Live single cells were gated, and the fluorescence intensities of cleaved and uncleaved CCF4 were excited with the 405 nm laser and detected with 450 nm and 535 nm emission filters at a BD LSR Fortessa. Results are presented as the mean fluorescence intensity (MFI) ratio between 450 and 535 nm emission filters. Fold change of percentage of translocation was calculated by comparing pimozide-stimulated versus non-stimulated cells. Additionally, 0.5 mM L-leucyl-L-leucine methyl ester (LLOMe) and 20 μM PR-619 (#T7739 and #T1862 respectively, TargetMol, Boston, USA) were used as negative and positive controls.

### Antigen processing assays

Endocytic capacity of DCs was assessed using Alexa Fluor™ 647 ovalbumin (OVA-AF647, #O34784, Thermo Fisher Scientific Inc.). DCs (2x10^5^ cells/well in ps-U-bottom-96-well plates) were pulsed with 0.25 µg/mL OVA-AF647 in the presence of 15 µM pimozide for 30, 60, and 90 min at 37 °C. Then flow cytometry staining was performed and Mean Fluorescence Intensity at AF647 channel was quantified in OVA-AF647^+^CD11c^+^ MHCII^+^ cells.

To measure acidification capacity, DCs (2x10^5^ cells/well in ps-U-bottom-96-well plates) were pulsed with 1 mg/mL of OVA coupled to a pH-sensitive dye pHrodo™ Red (#P36011, Thermo Fisher Scientific) and 1 mg/mL OVA-AF647 (a pH-insensitive dye as reference) for 15 min at 37 °C. After washing, cells were incubated with 15 µM pimozide or 15 µM Chloroquine (CQ, #C6628, Sigma Aldrich) for 30, 60, and 90 min. Flow cytometry staining was performed to measure acidification capacity by quantifying OVA-pHrodo MFI/OVA-AF647 MFI ratio in the double positive OVA-pHrodo^+^ OVA-AF647^+^ population.

For DQ-OVA processing assay, DCs (2x10^5^ cells/well in ps-U-bottom-96-well plates) were pulsed 15 min with 50 µg/mL of DQ™-OVA (#D12053, Thermo Fisher Scientific Inc., prepared as indicated by manufacturer). After pulse, cells were washed, incubated with 15 µM pimozide for 30, 60, and 90 min and antigen processing capacity was measured by quantifying DQ™-OVA MFI by flow cytometry.

### ROS production

Intracellular ROS production was quantified in DCs. DCs (2x10^5^ cells/well in ps-U-bottom-96-well plates) were treated with 15 µM pimozide for 2 h at 37 °C. 0.03% H_2_O_2_ (#805, Cicarelli Laboratorios, Santa Fe, Argentina) for 30 min was used as a positive control. When indicated, cells were pretreated for 30 min with the ROS scavenger NAC (10 mM) or the NADPH oxidase inhibitor DPI (10 µM) prior to pimozide stimulation.

After treatment, cells were washed and incubated with 1 µM 2′, 7′-dichlorodihydrofluorescein diacetate (H_2_-DCFDA; #D399, Thermo Fisher Scientific) for 30 min at 37 °C in the dark. Cells were subsequently washed, stained for surface markers to identify DC subsets, and analyzed by flow cytometry. ROS production was quantified as MFI of H_2_-DCFDA within total cDCs, cDC1, and cDC2 populations.

### Endosomal damage detection by Galectin-3 microscopy

Endosomal membrane damage was assessed by Galectin-3 redistribution. DCs (2x10^5^ cells/well in ps-flat-bottom-96-well plates) were incubated with 15 µM pimozide for 2 h at 37 °C. 0.5 mM LLOMe was used as a positive control for endolysosomal permeabilization. When indicated, cells were pretreated with NAC (10 mM) or DPI (10 µM) for 30 min prior to pimozide exposure.

Cells were then fixed, permeabilized, and stained with anti-Galectin-3 antibody. Nuclei were counterstained with Hoechst dye (#H3569; Life Technologies). Images were acquired using a 60× objective on InCell Analyzer 2500 HS, General Electric. Endosomal damage was quantified by measuring Galectin-3 puncta formation per cell using image analysis software.

### T cell proliferation assays

DCs (1.5 × 10^5^ cells/well in ps-U-bottom-96-well plates) were incubated with 0.25 mg/mL OVA and stimulated with 15 µM pimozide or with 2 μg/mL of fraction C of *Quillaja saponaria* bark (Desert King^®^, CA, USA) for 1.5 h at 37 °C. Cells were then washed and co-cultured with naïve CD8^+^ OT-I T cells (1.5 × 10^5^ cells/well) purified from spleens of OT-I mice by negative selection (EasySep Mouse CD8^+^ T Cell Isolation Kit, #19853A, StemCell Technologies, Vancouver, Canada), as previously described (53). Prior to co-culture, T cells were labeled with Cell Proliferation Dye eFluor™ 670 (eF670, #65-0840-90, eBioscience™, Thermo Fisher Scientific) by incubation in PBS containing either 0.3 µM or 3 µM dye, depending on the experiment, for 10 min at 37 °C protected from light. Cells were then washed with PBS and kept on ice until use. Concanavalin A (5 µg/mL; #C2010, Sigma-Aldrich) was used as a positive control for T cell proliferation and activation. After 72 h, culture supernatants were collected for IFN-γ quantification by ELISA. Cells were harvested, stained with Fixable Viability Dye eFluor™ 780 together with FITC anti-CD3, PE-Cy7 anti-CD11c, and PE-Cy5 anti-CD44 antibodies, and analyzed using Attune™ NxT or FACSCanto™ II flow cytometers.

### *In vivo* cytotoxic response

*In vivo* cytotoxic response was assayed as previously reported (54). Briefly, 1.0x10^6^ Flt3L-BMDCs from C57BL/6 mice were incubated with 10 mg/mL OVA with pimozide (15 µM) or with 2 μg/mL of fraction C of *Quillaja saponaria* bark (Desert King^®^, CA, USA) for 1.5 h at 37 °C and then were extensively washed with PBS. DCs were resuspended in saline solution and injected i.v. into C57BL/6 mice. Control mice received DCs without antigen.

WT splenocytes were incubated with SIINFEKL peptide (S8^+^ target cells) at 10 μg/mL for 30 minutes at 37 °C. S8^+^ cells were labeled with 3 μM eF670 and mixed in a 1:1 ratio with S8^-^ splenocytes (non target cells) labeled with 0.3 μM of the same intravital colorant. 100 μL of the cellular suspension containing approximately 10 x 10^6^ cells were injected i.v. to immunized mice at day 7 after immunization. The number of eF670^+^ cells remaining in the spleen after 24 h was determined by flow cytometry. Cytotoxicity was expressed as the percentage of lysis, calculated from [1- (R_control_/R_immunized_)] x 100], where R is given by the expression of % S8^-^ eF670^low^/% S8^+^ eF670^high^ cells for control and immunized mice, respectively.

### *In vivo* cross-priming Assay - CD8^+^ T cell specific effector function

DCs (1x10^6^ cells/well) were pulsed with OVA in the presence or absence of 15 µM pimozide for 1.5 h, washed and injected i.v. into C57BL/6 mice as described above. After 7 days post immunization, spleens were harvested and splenocytes were restimulated with 2 µg/mL SIINFEKL in the presence of Monensin and anti-CD107a for 5 h. After staining, CD8^+^ CD107a^+^ IFN-γ^+^ were quantified by flow cytometry. Moreover, after 48 h of *in vitro* peptide stimulation with 1 µg/mL SIINFEKL, tetramer staining was performed as described previously and H-2K^b^-SIINFEKL^+^ CD8^+^ CD3^+^ cells were analyzed by flow cytometry.

### Cytokine quantification

Cytokines were quantified in culture supernatants of DCs treated for 12 hs with 15 µM pimozide or 5 µg/mL CpG-ODN 1826, using the LEGENDplex Mouse Inflammation Panel 13-plex (#740150, BioLegend, San Diego, USA) according to manufacturer’s instructions. This panel allows simultaneous quantification of 13 mouse cytokines, including IL-1α, IL-1β, IL-6, IL-10, IL-12p70, IL-17A, IL-23, IL-27, MCP-1, IFN-β, IFN-γ, TNF-α,and GM-CSF.

IFN-γ production was measured by standard sandwich ELISA in culture supernatants of T cell proliferation assays *in vitro* and *ex vivo* stimulation of immunized mice splenocytes with 1 µg/mL SIINFEKL. IL-1β was determined by standard sandwich ELISA in culture supernatants of DCs stimulated for 18 h with 0.1 ug/mL LPS (from Escherichia coli, strain 0111:B4, Sigma Aldrich), 4 mM ATP (Sigma Aldrich) or with 0.1 ug/mL LPS for 3h alone plus 4 mM ATP for the rest of the incubation time. Capture and detection antibodies used in these assays are listed in **Supplementary Table 2**.

### Western blot analysis

DCs (2×10^6^) were treated with OVA in presence of 15 µM pimozide, J558 cell-supernatant containing 50 ng/mL GM-CSF or 30 µM STAT5-IN-1 (#T4216, TargetMol) at 37 °C for 4 hs. Total BMDCs were lysed in Sample buffer (0.35 M Tris-HCl, 0.63%V/V Glycerol, 10% P/V SDS, 0.012% P/V Bromofenol Blue, 2ß-mercaptoethanol). Equal lysate amounts were separated on a 12% acrylamide SDS–PAGE gel and transferred to a PVDF membrane. Blots were incubated with mouse monoclonal anti-actin as loading control (AC-40 clone, #A4700, Sigma Aldrich), rabbit monoclonal anti-STAT5 (D2O6Y clone, #94205, Cell Signaling Technology, Danvers, USA) and rabbit monoclonal anti-pSTAT5 (C11C15 clone, #9359, Cell Signaling) antibodies. Signals were detected with secondary antibodies stained with IRDye 800CW or IRDye 680CW (LI-COR Biosciences, NE, USA) using quantitative infrared fluorescence detection with Odyssey (LI-COR Biosciences). Blots were imaged in both the 700 and 800 nm channels by a single scan.

### Statistics

Statistical analyses were performed using GraphPad Prism (GraphPad 8.0.2 Software, CA, USA). Data are presented as mean ± SEM unless otherwise indicated. The specific statistical test employed for each experiment is indicated in the corresponding figure legend. Differences were considered statistically significant at p<0.05. For statistically non-significant differences (p>0.05), p values are indicated.

### Artificial intelligence generated content tools

During preparation of the final version of the manuscript, the authors used Claude (Anthropic), a large language model-based AI assistant, solely as a tool to improve the quality of the written English, check grammar, and refine the clarity and coherence of the text. The scientific content, analysis, and conclusions of the manuscript were entirely conceived and written by the authors. ChatGPT (v3.5) was used to generate illustrations of the experimental design.

## Results

### Pimozide improves antigen cross-presentation in conventional dendritic cells and potentiates CD8^+^ T cell responses *in vitro*

We first examined whether pimozide could influence the ability of DCs to present exogenous antigens on MHC I. To address this, we evaluated cross-presentation of chicken egg albumin (OVA) using the B3Z assay, a sensitive readout for H-2K^b^-SIINFEKL presentation. DCs pulsed OVA and treated with different doses of pimozide, induced markedly higher activation of B3Z cells compared with untreated controls, indicating that pimozide enhances access of antigens to the MHC I pathway in DCs (**Figure 1a**). Notably, this increase occurred without compromising DC viability (**Supplementary Figure S1a**). We next assessed whether pimozide-treated DCs could enhance the activation of naive antigen-specific CD8^+^ T cells. CD8^+^ T cells purified from the spleen of OT-I mice were co-cultured with antigen-pulsed DCs (gating strategy in **Supplementary Figure S1b**). Consistent with the hybridoma results, T cells co-cultured with pimozide-treated DCs showed more robust proliferation, higher CD44 expression (**Figures 1b, c**) and increased IFN-γ production (**Figure 1c**).

**Figure 1:**
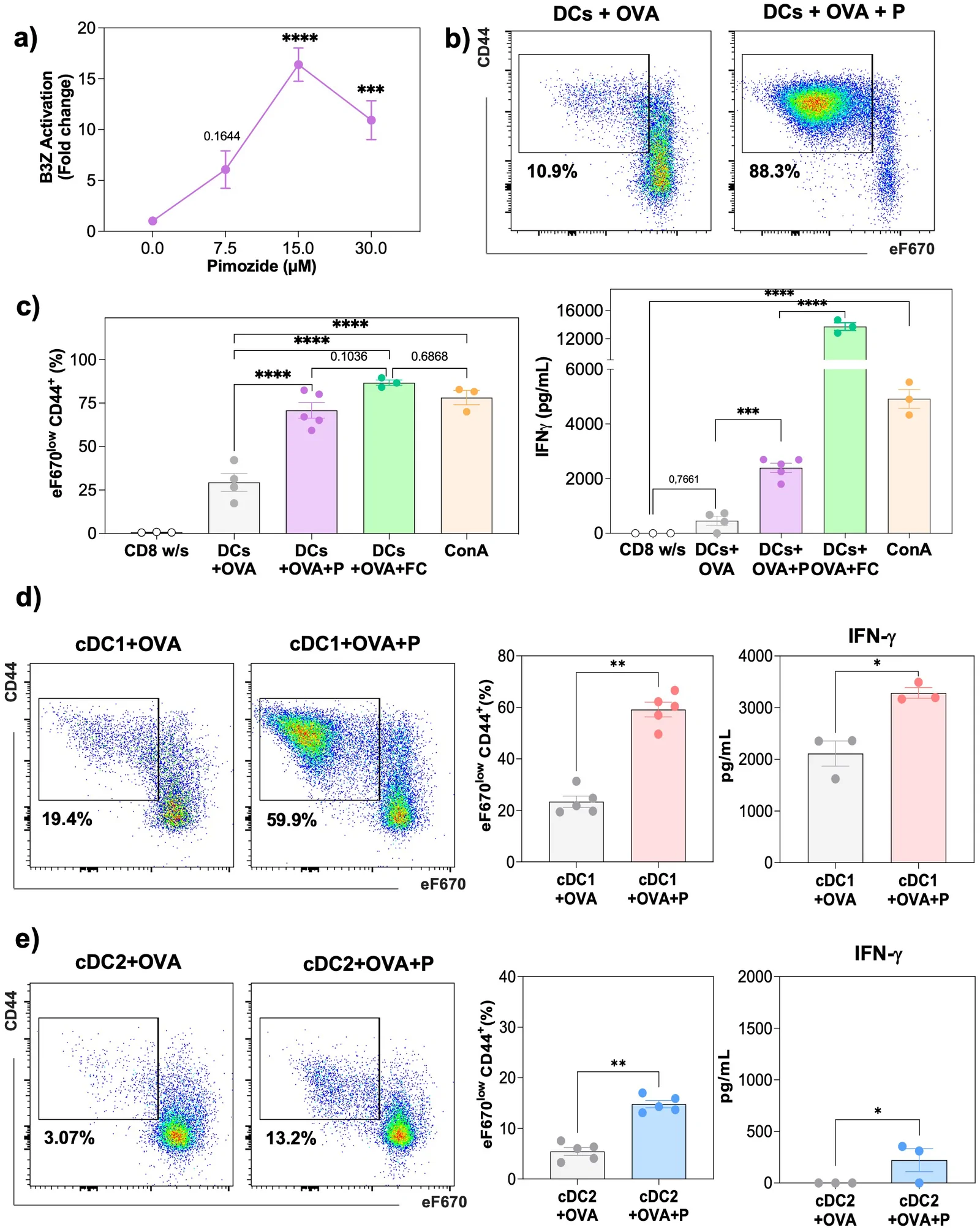
Pimozide enhances antigen cross-presentation and promotes antigen-specific CD8^+^ T cell activation by DCs *in vitro*. **(a)** DCs were incubated with 7.5, 15 or 30 µM pimozide (P) and 1 mg/mL OVA for 5 h at 37 °C. Then, cells were extensively washed and co-cultured with B3Z hybridoma cells for 18 h. B3Z activation was assessed using a colorimetric assay. Data are shown as fold change of absorbance relative to the condition without (P) (**b–e)** DCs (1.5 × 10^5^ cells/well) were incubated with 0.25 mg/mL OVA and stimulated with 15 µM P for 1.5 h at 37 °C. DCs were then co-cultured with CD8^+^ T cells (1.5 × 10^5^ cells/well) purified from OT-I mice, previously labeled with Cell Proliferation Dye eF670. After 72 h, cells were harvested, stained with specific antibodies and analyzed by flow cytometry. Concanavalin A (ConA; 5 μg/mL) and fraction C (FC, 2 μg/mL) of *Quillaja saponaria* bark were used as a positive control for T cell activation with total DCs (CD8^+^ w/s: OT-I purified CD8^+^ T cells without stimulus). **(b)** Representative dot plot analysis showing eF670 fluorescence intensity against CD44 expression. **(c)** Frequency of eF670^low^ CD44^+^ T cells and IFNγ concentration in culture supernatants. **(d, e)** Representative dot plot, frequency of eF670^low^ CD44^+^ CD8^+^ T cells and IFN-γ concentration in culture supernatants are shown for **d**) cDC1 (CD11c^+^ MHCII^+^ CD24^high^ SIRPα^-^, and **(e)** cDC2 (CD11c^+^ MHCII^+^ SIRPα^high^). Statistical analysis in **(a, c)** was conducted using one-way ANOVA with Tukey’s multiple comparisons test. Statistical analysis in **(d–e)** was conducted using unpaired t-test relative to the condition without P. Data are presented as mean ± SEM from at least two independent experiments (n≥3). Significance levels: p<0.05 (*), p<0.01 (**), p<0.001 (***), and p<0.0001 (****). Non-significant differences (p>0.05) are indicated with their corresponding p values.

Because cDC1 and cDC2 subsets differ in their intrinsic ability to cross-present antigens, we evaluated whether the effect of pimozide was restricted to a particular subset. To this end, Flt3L-derived DCs were sorted into cDC1 and cDC2 subpopulations (gating strategy in **Supplementary Figure S1c**), treated with pimozide, and subsequently co-cultured with OVA and CD8^+^ T cells from OT-I mice to assess cross-presentation as described above. cDC1 cells showed a strong enhancement in their ability to activate OT-I cells following treatment with pimozide (**Figure 1d**). Interestingly, cDC2 cells—which typically exhibit lower antigen cross-presentation efficiency—also responded to pimozide with increased T cell proliferation and IFN-γ secretion (**Figure 1e**), although to a much lesser extent than cDC1s.

Together, these findings demonstrate that pimozide enhances antigen cross-presentation by targeting cellular pathways shared in both cDCs subsets and strengthens downstream CD8^+^ T cell activation, supporting our hypothesis that this compound can modulate DC physiology to potentiate CD8^+^ T cell responses.

### Pimozide enhances the surface display and stability of H-2K^b^–SIINFEKL complexes on dendritic cells

Since pimozide enhances antigen cross-presentation and CD8^+^ T cell activation, we next examined whether it also modulated the expression of surface molecules involved in antigen presentation and T cell priming. CpG-ODN was included as a positive maturation control. After 12 h of treatment, DCs exposed to pimozide displayed increased levels of MHC II and clear upregulation of the costimulatory molecules CD80 and CD86, although to a lesser extent than with CpG-ODN (**Figure 2a**). In contrast, no changes in cytokine secretion were observed under the same conditions (**Supplementary Figure S1d**).

**Figure 2:**
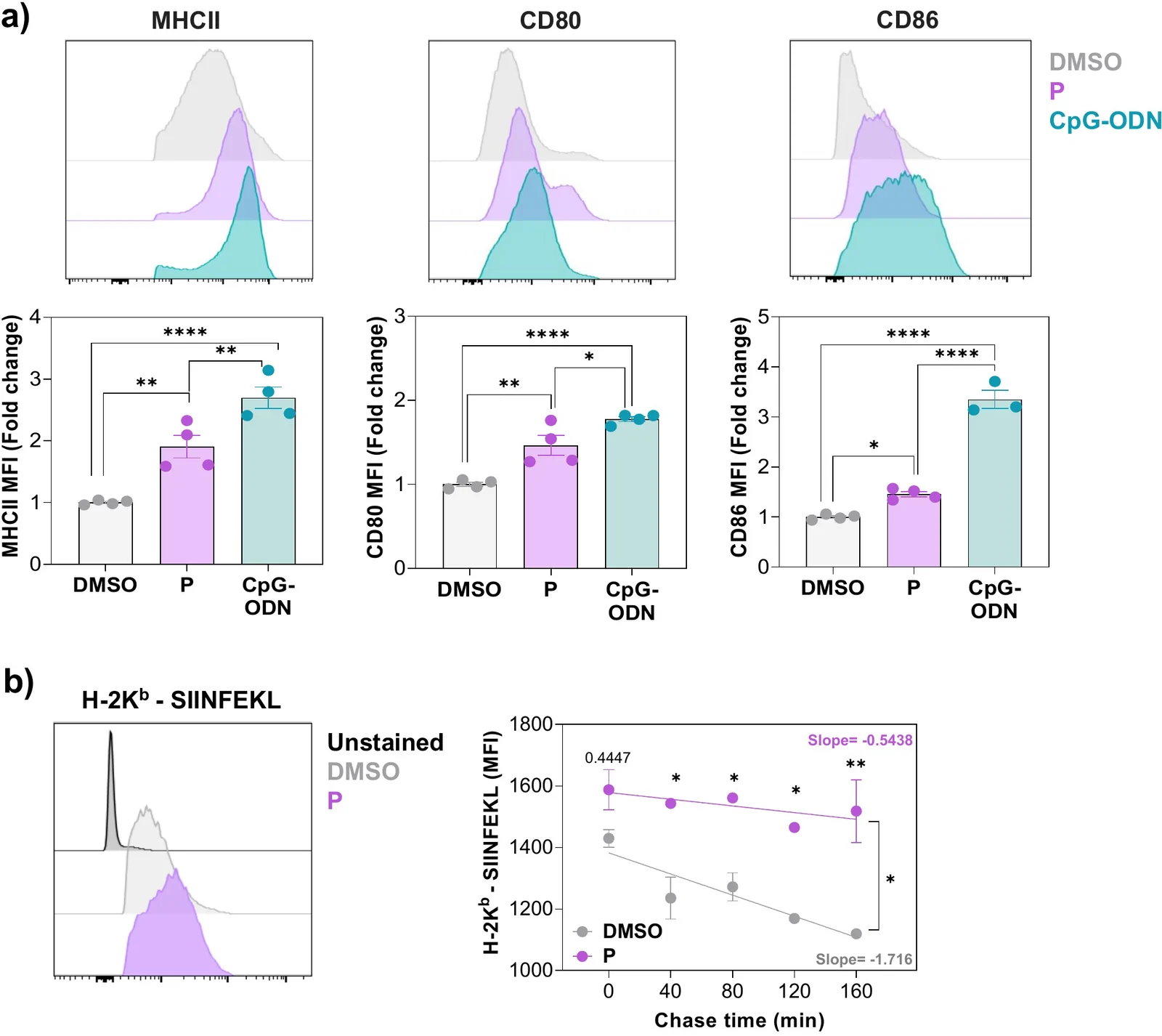
Pimozide enhances the surface display and stability of H-2Kb–SIINFEKL complexes on dendritic cells. **(a)** DCs were incubated with 15 µM pimozide (P) or 5 µg/mL CpG-ODN for 12 h at 37 °C. Cells were then stained with specific antibodies against costimulatory molecules and analyzed by flow cytometry. Representative histograms (top) and quantification (bottom) of MHCII, CD80 and CD86 expression reported as MFI on CD11c^+^ MHCII^+^ cells are shown. **(b)** DCs were pulsed with 100 ng/mL SIINFEKL peptide in the presence or absence of 15 µM P for 15 min, extensively washed, stained with an anti-H-2K^b^-SIINFEKL antibody, chased for the indicated times with a secondary anti-IgG antibody and analyzed by flow cytometry. Left: representative histograms at t=160 min of DCs under basal conditions (empty), incubated with SIINFEKL alone (grey) or plus P (purple). Right: frequency of DCs expressing surface H-2K^b^-SIINFEKL complexes. Statistical analysis in **(a)** was conducted using one-way ANOVA with Tukey’s multiple comparisons test. Statistical analysis in **(b)** was performed using linear regression to obtain the slopes of the curves in each experimental condition and two-way ANOVA with Tukey’s multiple comparisons test. Data are presented as mean ± SEM from two independent experiments (n≥3). Significance levels: p<0.05 (*), p<0.01 (**), and p<0.0001 (****). Non-significant differences (p>0.05) are indicated with their corresponding p values.

Because efficient priming of CD8^+^ T cells relies on the sustained surface expression of peptide–MHC I complexes, we evaluated whether pimozide affects the stability of H-2K^b^–SIINFEKL complexes on the DC surface. DCs were pulsed with the SIINFEKL peptide, and the decay of surface complexes was monitored over time using the monoclonal antibody 25-D1.16, which specifically recognizes the H-2K^b^-SIINFEKL complex. DCs treated with pimozide exhibited higher levels of H-2K^b^-SIINFEKL on DC surface from 40 min to 160 min of chase and maintained these complexes on their surface for a longer period compared with untreated cells (**Figure 2b**). Linear regression analysis confirmed a reduced decay rate of H-2K^b^–SIINFEKL complexes on pimozide-treated DCs, indicating that pimozide enhances the stability of peptide–MHC I complexes at the cell surface (**Figure 2b**).

Together, these findings show that pimozide increases the expression of co-stimulatory molecules and enhances the surface display and persistence of H-2K^b^-SIINFEKL complexes on the DCs.

### Pimozide-treated dendritic cells exhibit improved antigen translocation capacity and reduced endolysosomal acidification

Cross-presentation can proceed through two major routes: a vacuolar pathway, in which antigens are processed entirely within endolysosomal compartments, and a cytosolic pathway, in which internalized antigens are partially processed within endolysosomal compartments, escape to the cytosol for proteasome-mediated degradation, and re-enter the endolysosomal system for final peptide loading onto MHC I molecules (4). Because pimozide enhanced surface display of H-2K^b^–SIINFEKL complexes and CD8^+^ T cell activation, we investigated which cross-presentation pathway was engaged upon pimozide treatment by assessing whether OVA processing was proteasome-dependent. DCs were stimulated in the presence of lactacystin, a specific proteasome inhibitor that blocks the cytosolic route. In the presence of the proteasome inhibitor, pimozide-treated DCs completely abolished their strengthened cross-presentation capacity, evidenced by a significant decrease in B3Z activation compared to the untreated control (**Figure 3a**). This finding demonstrates that the enhanced cross-presentation induced by pimozide is proteasome-dependent, indicating that pimozide promotes one or more steps involved in the cytosolic pathway of antigen cross-presentation, possibly by facilitating antigen escape from endosomes to the cytosol or by altering endosomal conditions that favor antigen translocation.

**Figure 3:**
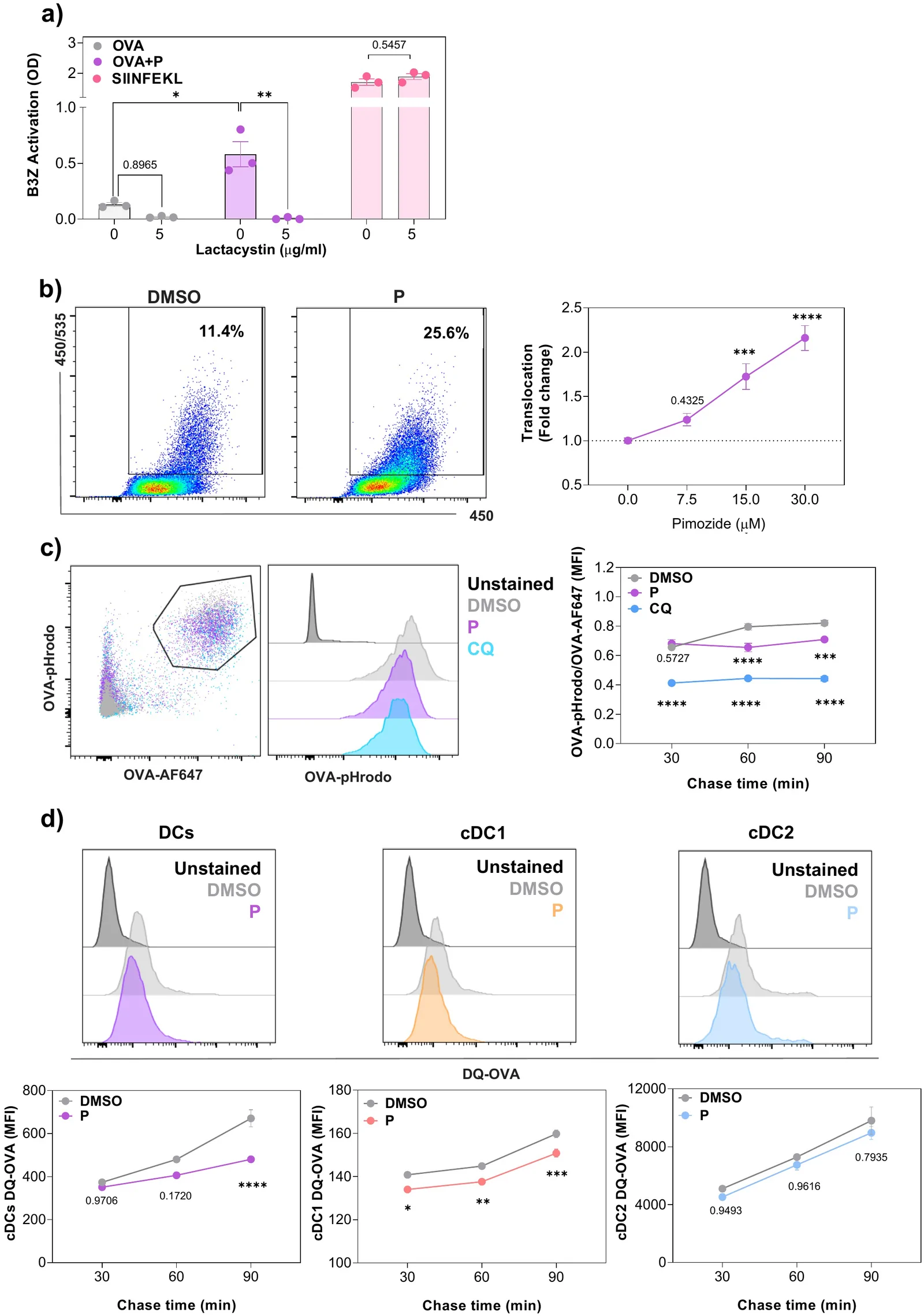
DCs treated with pimozide show improved antigen translocation and reduced endosomal acidification. **(a)** DCs were pretreated with 5 µg/mL lactacystin for 30 min and subsequently incubated with 15 µM pimozide (P) and 1 mg/mL OVA for 5 h at 37 °C. Cells were then extensively washed and co-cultured with B3Z hybridoma cells for 18 h. B3Z activation was assessed using a colorimetric assay. **(b)** To evaluate antigen translocation into the cytosol, JAWS II- DCs were pulsed with β-lactamase at 37 °C. After 30 min, 15 µM P was added for 2 h. Cells were washed, loaded with the CCF4-AM substrate for 1.5 h, stained with a viability marker, and analyzed by flow cytometry. Left: representative dot plot analysis showing the frequency of cells undergoing antigen translocation. Right: quantification of antigen translocation, expressed as fold change in the percentage of cells relative to unstimulated DCs. **(c)** To measure endosomal acidification, DCs were pulsed with 1 mg/mL of OVA-pHrodo Red and 1 mg/mL OVA-Alexa Fluor 647 for 15 min, washed, incubated with 15 µM P or 15 µM chloroquine (CQ) for the indicated chase times prior, and analyzed by flow cytometry. Left: representative histogram overlays and dot plots of pHrodo and AF-647 fluorescence intensity in the different groups. Right: relative endosomal acidification calculated as the pHrodo: AF647 MFI ratio. **(d)** To assess antigen processing capacity, DCs were pulsed with 50 µg/mL DQ™- OVA for 15 min at 37 °C, washed, and incubated for 30, 60, or 90 min in the presence of 15 µM P or vehicle. Cells were stained with specific antibodies to identify total DCs (left), cDC1s (middle), and cDC2s (right) subsets. Top panels show representative histogram overlays of DQ™-OVA fluorescence (DCs not incubated with DQ™-OVA, “unstained”, were used as baseline signal). Bottom graphs show the quantification of DQ™-OVA MFI. For all panels, data are presented as mean ± SEM from at least two independent experiments (n≥3). Statistical analysis was conducted using two-way ANOVA with Tukey’s multiple comparisons test, except in **(b)**, where one-way ANOVA with Tukey’s multiple comparisons test was applied. Significance levels: p<0.05 (*), p<0.01 (**), p<0.001 (***), and p<0.0001 (****). Non-significant differences (p>0.05) are indicated with their corresponding p values.

In the cytosolic pathway of antigen cross-presentation, antigens are translocated across the endosomal membrane into the cytosol. Although these mechanisms are not fully defined, they are thought to involve membrane destabilization, ROS–mediated lipid peroxidation, and components of the ER-associated degradation (ERAD) machinery (6, 10, 11, 55–58). Once in the cytosol, antigens are degraded by the proteasome, and the resulting peptides are transported by TAP into the endoplasmic reticulum—or back into endosomal compartments—for loading onto MHC I molecules (4). This endosome-to-cytosol escape step is considered a critical and rate-limiting event in efficient antigen cross-presentation by DCs through the cytosolic pathway. To directly evaluate whether pimozide affects antigen escape from endosomes, we used the β-lactamase/CCF4 assay modified from Cebrián et al. (52). For this assay, we employed the JAWSII dendritic cell line, which offers improved sensitivity over primary BMDCs due to its greater cellular homogeneity. Notably, pimozide also enhanced antigen cross-presentation in these cells (**Supplementary Figure S2a**). JAWSII cells treated with pimozide showed a higher fraction of cells in which β-lactamase reached the cytosol compared with untreated cells, in a dose-dependent manner (**Figure 3b**), indicating that pimozide facilitates antigen translocation from endosomes to the cytosol in DCs. PR-619, an inhibitor of deubiquitinases that likely impairs antigen translocation by disrupting ubiquitin-dependent regulation of ERAD-like retrotranslocation machinery and p97/VCP-mediated extraction (44, 59), was used as a negative control, while L-leucyl-L-leucine methyl ester (LLOMe), a lysosomotropic agent that induces endolysosomal permeabilization (6, 60, 61), served as a positive control (**Supplementary Figure S2b**). Given that endosomal permeabilization has been associated with inflammasome activation in DCs, we investigated whether the NLRP3 inflammasome contributed to the enhancement of cross-presentation induced by pimozide. To address this, DCs generated from NLRP3-deficient mice were pulsed with OVA in the presence or absence of pimozide and subsequently co-cultured with OT-I CD8^+^ T cells. Pimozide enhanced cross-presentation to a similar extent in both wild-type and NLRP3-deficient DCs, as shown by comparable CD8^+^ T cell activation levels (**Supplementary Figure S3a**), confirming the functional validity of the knockout (**Supplementary Figure S3b**). Furthermore, pimozide did not induce IL-1β secretion in DCs (**Supplementary Figure S1b**), collectively indicating that the enhancement of cross-presentation by pimozide operates independently of NLRP3 inflammasome activation.

Efficient cross-presentation critically depends on the maintenance of a mildly acidic endosomal environment that preserves antigen integrity while allowing controlled cytosolic access (8, 62, 63). To address this, we next evaluated whether pimozide altered endosomal acidification in DCs. Using OVA-pHrodo as a pH-sensitive probe and OVA-AF647 as a pH-independent reference, we observed that pimozide-treated DCs displayed significantly lower pHrodo: AF647 ratios than untreated cells (**Figure 3c**), indicating a less acidic endosomal environment. This effect resembled that induced by chloroquine, a well-established inhibitor of endosomal acidification (43), though it was less pronounced. Together, these observations suggest that pimozide impairs the conversion of endosomes into fully acidic degradative compartments, potentially creating endosomal conditions more favorable for antigen cross-presentation.

Because endosomal pH directly influences antigen degradation, we next examined whether pimozide affected proteolytic processing within endosomal compartments using the DQ™-OVA probe, a self-quenched substrate that fluoresces upon proteolytic degradation (62–65). In total DC populations, pimozide treatment resulted in a progressively lower DQ-OVA fluorescence over time compared with control cells, indicating decreased antigen degradation (**Figure 3d**, left). We then examined whether this reduction was restricted to a particular DC subset. When analyzed separately, cDC1 displayed a clear reduction in DQ-OVA fluorescence upon pimozide treatment (**Figure 3d**, middle), whereas no significant changes were observed in cDC2 at any of the analyzed time points (**Figure 3d**, right). These results indicate that pimozide selectively limits antigen degradation in cDC1, the DC subset specialized in cross-presentation. These results could not be attributed to enhanced antigen internalization, as pimozide-treated DCs exhibited similar OVA-AF647 internalization kinetics to those of untreated controls at all analyzed time points (**Supplementary Figure S3c**). This indicates that the effects of pimozide on antigen translocation and proteolytic processing occur downstream of endocytosis, at the level of endosomal processing and trafficking.

Together, these findings demonstrate that pimozide reshapes antigen-processing and trafficking events in DCs by attenuating endosomal acidification, limiting antigen degradation, and facilitating antigen escape from endosomes to the cytosol. These coordinated effects create an endosomal environment more conducive to cross-presentation, thereby providing a mechanistic basis for the enhanced CD8^+^ T cell activation elicited by pimozide-treated DCs.

### Pimozide induces DCs endosomal damage that can be reverted by ROS inhibition

Previous studies demonstrated that controlled ROS production by NOX2 is a key mechanism for alkalinizing endocytic compartments through proton consumption, thereby limiting excessive antigen degradation and promoting endosomal membrane permeabilization, which facilitates antigen escape into the cytosol (8, 9, 56, 57, 62). As pimozide-treated DCs exhibited reduced endosomal acidification and enhanced antigen access to the cytosol, we investigated whether these effects were associated with increased ROS generation. Intracellular ROS production was quantified using the H_2_-DCFDA probe, a cell-permeable oxidation-sensitive fluorescent dye widely used to estimate global ROS levels, with hydrogen peroxide included as a positive control for assay validity. Pimozide treatment for 2 h induced a clear increase in ROS levels in DCs compared with DMSO-treated controls (**Figure 4a**), with similar results observed in both cDC1 and cDC2 populations (**Figure 4b**). These findings indicate that pimozide triggers a measurable oxidative response in DCs, consistent with modulation of endosomal acidification and antigen translocation.

**Figure 4:**
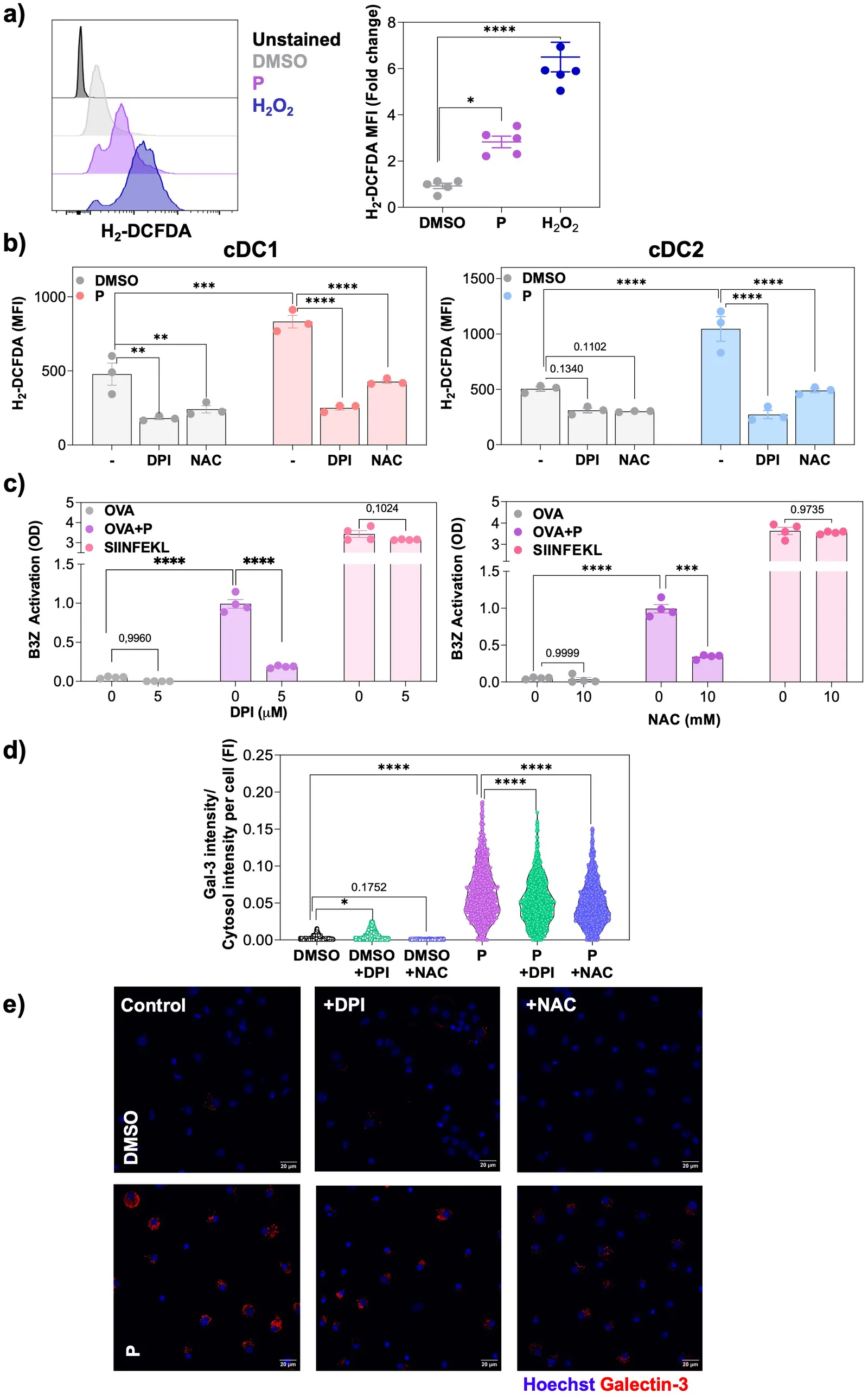
Pimozide induces DCs endosomal damage that is reversed by ROS inhibition. **(a)** DCs were incubated 15 µM pimozide (P) for 2 h or 0.03% H_2_O_2_ for 30 min at 37 °C. Then, cells were incubated with 1 µM H_2_-DCFDA probe for 30 min at 37 °C. Representative histogram overlays, and quantification of ROS production in CD11c^+^ MHCII^+^ cells, expressed as fold change in H_2_-DCFDA MFI relative to DMSO-treated controls. **(b)** DCs were incubated with 10 µM DPI or 10 mM NAC for 20 min at 37 °C, followed by addition of 15 µM (P) After 2 h, cells were loaded with 1 µM H_2_-DCFDA probe for 30 min and stained for DC subset identification. Analysis was performed on cDC1 (left) and cDC2 (right) populations. ROS production was quantified as H_2_-DCFDA MFI by flow cytometry. **(c)** DCs were pretreated with 10 µM DPI (left panel) or 10 mM NAC (right panel) for 20 min, followed by incubation with 15 µM P and 1 mg/ml OVA or 10 ng/ml SIINFEKL for 3 h at 37 °C. Cells were extensively washed and co-cultured with B3Z hybridoma cells for 18 h. B3Z activation was measured by a colorimetric assay. **(d, e)** DCs were incubated with 10 µM DPI or 10 mM NAC for 20 min and then treated with 15 µM P for 2 h at 37 °C. Cells were fixed, permeabilized and stained with PE anti-Galectin-3 and Hoechst. Galectin-3 staining was quantified as the ratio of total Gal-3 fluorescence intensity (FI) to cytosolic FI per cell **(d)**. Representative images acquired at 60X magnification are shown **(e)**. Statistical analysis was conducted using one-way ANOVA in panels **(a, d)** or two-way ANOVA for panels **(b–c)**, and **(d)**, followed by Tukey’s multiple comparisons test. Data are presented as mean ± SEM of at least two independent experiments (n≥3). Significance levels: p<0.05 (*), p<0.01 (**), p<0.001 (***), and p<0.0001 (****). Non-significant differences (p>0.05) are indicated with their corresponding p values.

To determine the source of pimozide-induced ROS, DCs were pretreated with diphenyleneiodonium (DPI), a flavoprotein-containing enzymes inhibitor, including NADPH oxidases (NOX family), commonly used to assess the contribution of NOX2-derived phagosomal ROS. Alternatively, DCs were pretreated with the antioxidant N-acetylcysteine (NAC) (8, 9, 62). NAC acts as a ROS scavenger and glutathione precursor (66) and is frequently used to evaluate redox-dependent mechanisms (67). Pretreatment with either compound significantly prevented the pimozide-induced increase in ROS levels in both cDC1 (**Figure 4b**, left) and cDC2 (**Figure 4b**, right) populations. These results indicate that pimozide-induced ROS production is largely dependent on NADPH oxidase activity, consistent with a NOX2-mediated mechanism.

We next assessed whether ROS production was required for the pimozide-enhanced antigen cross-presentation. Interference with ROS signaling using DPI or NAC markedly reduced pimozide-enhanced OVA cross-presentation (**Figure 4c**). Importantly, direct SIINFEKL peptide presentation remained unaffected by these treatments (**Figure 4c**), indicating that ROS are specifically required for the processing or translocation of antigens rather than for peptide loading onto MHC I molecules. These findings place ROS upstream of MHC I assembly, consistent with the NOX2-mediated modulation of endosomal acidification described above, and support a model in which pimozide-induced ROS generation drives the endosomal remodeling required for efficient cross-presentation.

Given the established role of ROS in promoting membrane destabilization, we next evaluated whether endosomal integrity was compromised under pimozide treatment. Galectin-3 is a cytosolic lectin that enters endocytic compartments after membrane damage, serving as a marker for membrane disruption (6, 60, 68). Thus, we performed Galectin-3 staining in pimozide-treated DCs, using LLOMe as a positive control for endosomal membrane damage (**Supplementary Figure S3d**). Notably, pimozide treatment significantly increased Galectin-3 accumulation in punctate foci compared to DMSO-treated cells, indicating that pimozide causes endolysosomal destabilization. Importantly, this effect was significantly attenuated by pretreatment with DPI or NAC (**Figures 4d, e**), confirming that pimozide-induced membrane damage is ROS-dependent.

Collectively, these results demonstrate that pimozide induces ROS production in DCs that contributes to endosomal alkalinization, reduced proteolytic activity, and partial membrane permeabilization - a coordinated remodeling of endosomal physiology that facilitates antigen escape into the cytosol.

### Pimozide inhibition of STAT5 results in enhanced antigen cross-presentation by DCs

Pimozide has been described in several cellular contexts as an inhibitor of STAT5 phosphorylation (31, 32, 37–41, 69). However, the role of STAT5 in DCs, particularly its potential involvement in antigen handling or cross-presentation, remains largely unexplored. Western blot analysis revealed that pimozide reduced both total STAT5 and p-STAT5 levels compared with OVA-treated controls (**Figures 5a, b**), indicating that pimozide interferes with STAT5 signaling in DCs. Granulocyte-macrophage colony-stimulating factor (GM-CSF) was included as a positive control of STAT5 activation (70, 71) (**Supplementary Figures S4a, b**). To determine whether direct pharmacological STAT5 inhibition affects cross-presentation efficiency, we used STAT5-IN-1, a small-molecule inhibitor that blocks STAT5 transcriptional activity by interfering with phosphorylation-dependent activation and dimerization (72), thereby preventing downstream gene transcription. We additionally evaluated ROS production following pharmacological STAT5 inhibition with STAT5-IN-1. Importantly, STAT5-IN-1 did not increase ROS production, despite its ability to enhance cross-presentation (**Supplementary Figure S4c**). DCs were pulsed with OVA in the presence of increasing doses of STAT5-IN-1, which resulted in a dose-dependent increase in B3Z activation (**Figure 5c**), demonstrating that pharmacological STAT5 inhibition enhances antigen cross-presentation. To determine whether these effects translated into enhanced T cell activation, DCs exposed to STAT5-IN-1 were co-cultured with naïve OT-I CD8^+^ T cells. STAT5-IN-1–treated DCs promoted significantly higher OT-I proliferation compared with control DCs (**Figure 5d**), indicating that reduced STAT5 activity enhances DC-driven CD8^+^ T cell responses.

**Figure 5:**
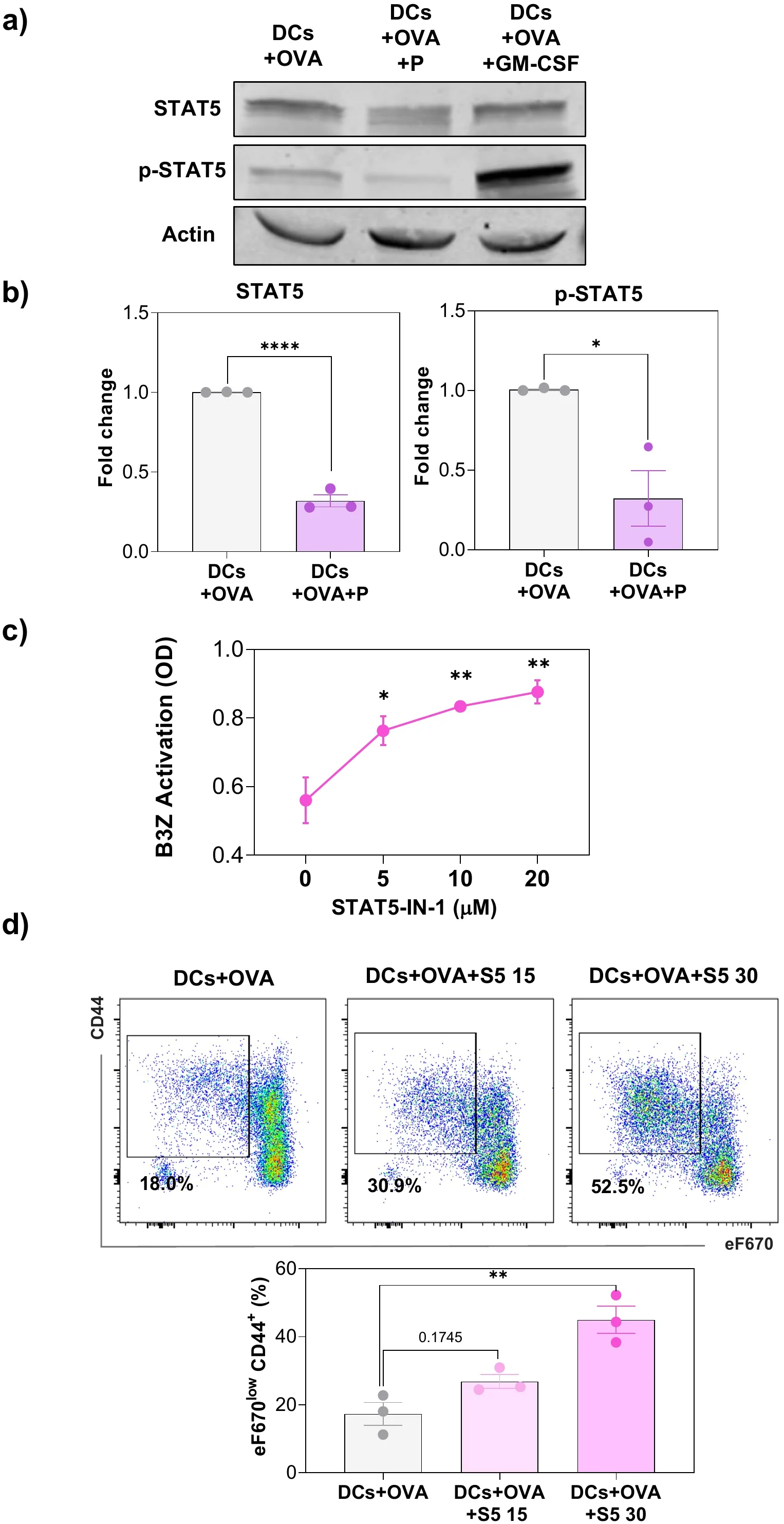
STAT5 signaling is implicated in pimozide-mediated cross-presentation. **(a, b)** DCs were incubated with 15 µM pimozide (P) or 50 ng/mL GM-CSF in the presence of 1 mg/ml OVA for 4 h at 37 °C. Cells were then washed and lysed. Representative Western Blot images **(a)** and quantification of STAT5 and phospho-STAT5 levels, expressed as fold change relative to OVA-treated DCs **(b)** are shown. **(c)** DCs were incubated with 1 mg/ml OVA with increasing concentrations of STAT5-IN-1 for 5 h at 37 °C. Cells were then washed and co-cultivated with B3Z hybridoma for 18 h. After lysis, B3Z activation was measured by a colorimetric assay. **(d)** DCs were incubated with 0.25 mg/ml OVA and stimulated with either 15 or 30 µM STAT5-IN-1 (S5) for 1.5 h at 37 °C. DCs were then co-cultured with CD8^+^ T cells purified from OT-I mice, previously labeled with Cell Proliferation Dye eF670. After 72 h, cells were harvested, stained with specific antibodies and analyzed by flow cytometry. Representative dot plot analysis (top) and quantification of eF670^low^ CD44^+^ CD8^+^ T cells (bottom) are shown. Statistical analysis in **(b)** was conducted using unpaired t-test relative to the condition without (P) Statistical analysis in **(c, d)** was conducted using one-way ANOVA and Tukey’s multiple comparisons test. Data are presented as mean ± SEM of at least two independent experiments (n≥3). Significance levels: p<0.05 (*), p<0.01 (**), and p<0.0001 (****). Non-significant differences (p>0.05) are indicated with their corresponding p values.

Together, these results identify STAT5 signaling as a previously unrecognized negative regulator of DC-mediated antigen processing and demonstrate that its pharmacological inhibition reproduces the major functional outcomes elicited by pimozide, including enhanced cross-presentation and increased CD8^+^ T cell activation.

### Immunization with pimozide-treated DCs induces functional, antigen-specific cytotoxic CD8^+^ T cells *in vivo*

To determine whether the enhancement of cross-presentation observed *in vitro* translated into improved CD8^+^ T cell immunity *in vivo*, we evaluated the ability of pimozide-treated DCs to elicit antigen-specific cytotoxic responses. DCs were pulsed with OVA in the presence or absence of pimozide, extensively washed, and adoptively transferred into naïve mice (**Supplementary Figure S5a**). We first assessed the effector function of CD8^+^ T cells by measuring degranulation and cytokine production following ex vivo re-stimulation with SIINFEKL. Splenocytes from mice immunized with pimozide-treated DCs exhibited a significantly higher frequency of CD107a^+^ IFN-γ^+^ double-positive CD8^+^ T cells than those from control groups (**Figure 6a**), indicating that pimozide enhances both cytotoxic potential and inflammatory cytokine production in antigen-specific CD8^+^ T cells. We next quantified the frequency of OVA-specific T cells using H-2K^b^–SIINFEKL tetramer staining (representative gating strategy in **Supplementary Figure S5c**). Immunization with pimozide-treated OVA-loaded DCs resulted in a robust and significantly greater expansion of tetramer-positive CD8^+^ T cells compared with mice receiving OVA-loaded DCs alone (**Figure 6b**), confirming that pimozide-treated DCs drive stronger antigen-specific CD8^+^ T cell responses *in vivo*. To directly assess whether these enhanced effector functions translated into antigen-specific cytotoxicity *in vivo*, we performed an *in vivo* killing assay (representative gating strategy in **Supplementary Figure S5d**). Seven days after immunization, mice that received pimozide-treated OVA-loaded DCs exhibited markedly increased specific lysis of SIINFEKL-pulsed target cells compared with mice immunized with OVA-loaded DCs alone (**Figure 6c**), demonstrating that pimozide-treated DCs promote the generation of functional CTLs capable of executing antigen-specific cytotoxic responses *in vivo*.

**Figure 6:**
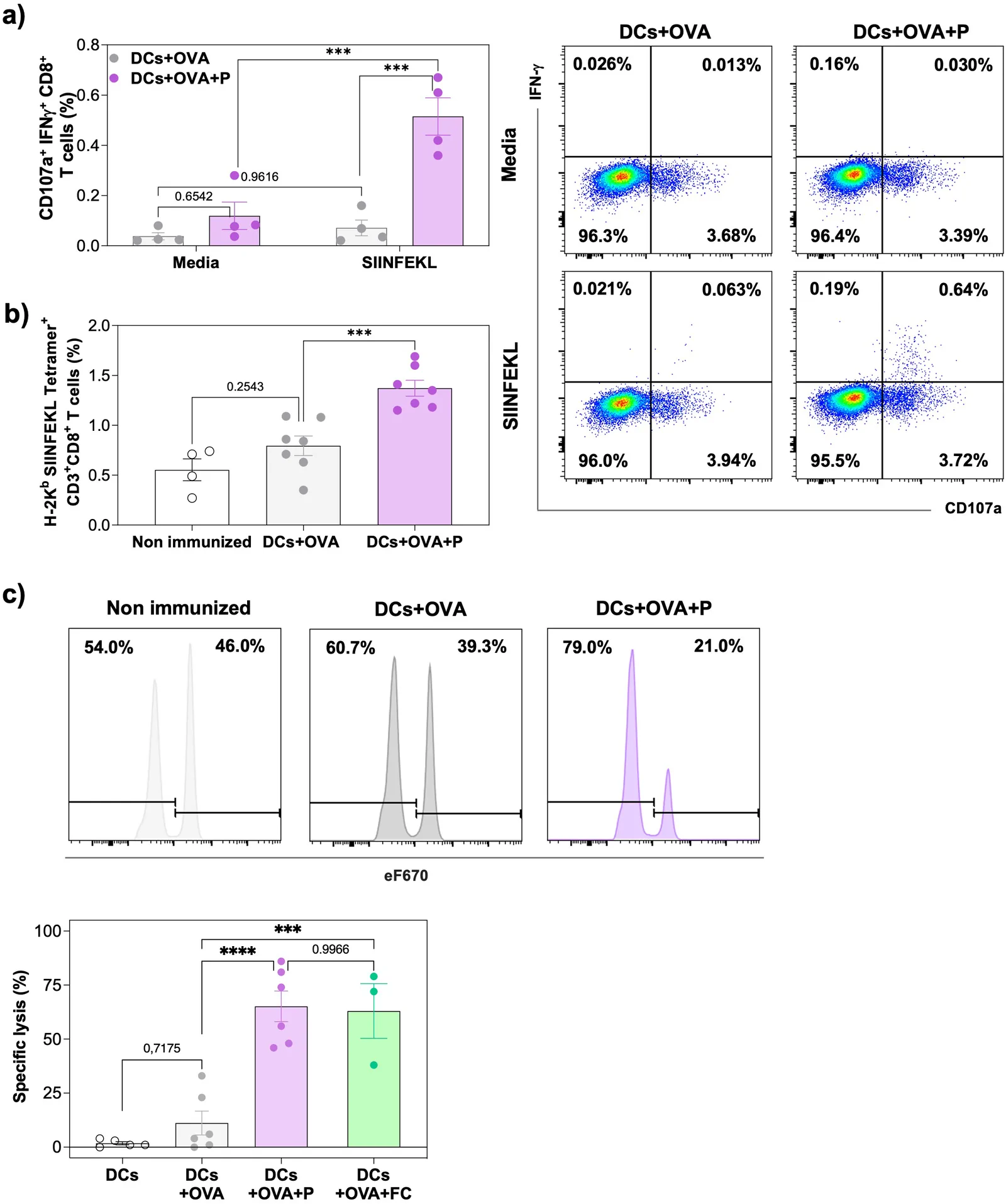
Immunization with pimozide-treated DCs induces functional and antigen-specific cytotoxic CD8^+^ T cells *in vivo.* DCs were incubated with 10 mg/mL OVA in the presence or absence of 15 µM pimozide (P, in a-c) or 2 μg/mL fraction C (FC) of *Quillaja saponaria* bark for 1.5 h at 37 °C (only in **c**). Then, cells were extensively washed and injected i.v. into naïve C57BL/6 mice. Seven days later, frequency of CD107^+^ IFN-γ CD8^+^ T cells **(a)** and expansion of SIINFEKL-specific CD8^+^ T cell population **(b)** in splenocytes as well as *in vivo* CTL response **(a)** were determined. **(a)** Seven days after immunization, splenocytes were harvested and restimulated with or without 2 µg/mL SIINFEKL peptide for 5 h in the presence of monensin and anti-CD107a antibody. Cells were then stained with antibodies for surface markers, fixed, permeabilized, stained with anti-IFNγ and analyzed by flow cytometry. Representative dot plots of IFNγ vs CD107a staining (right) and quantification of CD107a^+^ IFNγ^+^ cells within total CD8^+^ T cell population (left) are shown. **(b)** Seven days after immunization, splenocytes were stimulated with 1 µg/mL SIINFEKL peptide for 48 h. Then cells were stained with H-2K^b^-SIINFEKL tetramer and surface marker antibodies. **(c)** Seven days after immunization, an *in vivo* killing assay was performed. Target cell lysis was assessed by flow cytometry 24 h after target cell transfer. Representative histograms (top panel) and quantification (bottom panel) of specific target cell lysis. Statistical significance was determined by two-way **(a)** and one-way **(b, c)** ANOVA and Tukey’s multiple comparisons test. Data are presented as mean ± SEM of at least two independent experiments (n≥4). Significance levels: p<0.001 (***), and p<0.0001 (****). Non-significant differences (p>0.05) are indicated with their corresponding p values.

In summary, these *in vivo* findings provide the first physiological validation of our *in vitro* observations, confirming that pimozide treatment enhances the capacity of DCs to prime potent antigen-specific CD8^+^ T cell responses and to support the development of functional cytotoxic immunity.

## Discussion

Cross-presentation is dynamically influenced by the activation state of dendritic cells, and several studies have shown that antigen routing toward the MHC I pathway is tightly coordinated with dendritic cell maturation programs (73, 74). Accordingly, classical approaches to enhance cross-presentation have largely relied on innate immune stimuli that simultaneously promote strong inflammatory activity. We have previously demonstrated that PolyU is capable of boosting cross-presentation while inducing robust dendritic cell maturation and CD8^+^ T cell priming (53, 75). The same has been found for other Toll-like receptor (TLR) ligands (10, 76, 77). Beyond these classical innate stimuli, emerging evidence indicates that cross-presentation can also be enhanced through pharmacological modulation of intracellular antigen-handling pathways (12, 13, 44). Consistent with the concept that cross-presentation can be modulated independently of strong inflammatory activation, our results show that pimozide enhances dendritic cell cross-presentation while inducing only modest phenotypic maturation, characterized by a subtle increase in MHC class II, CD80, and CD86 expression without major changes in inflammatory cytokine production. This uncoupling has recent mechanistic precedent: in cDC1, DNGR-1 signaling actively limits dendritic cell activation while preserving efficient cross-presentation of dead-cell-associated antigens, indicating that these two processes can be functionally segregated at the level of receptor signaling (74). A comparable dissociation has been described for saponin-based adjuvants, which enhance antigen cross-presentation without inducing CD80 or CD86 upregulation (12), for carbomer-based adjuvants that engage cross-presentation through a distinct metabolic program (13), and for small molecules that promote antigen escape from endolysosomal compartments (44). In line with this precedent, fraction C of the *Quillaja saponaria* bark— the lower-activity, better-tolerated saponin component of the Matrix-M adjuvant — elicited antigen-specific T cell proliferation and *in vivo* cytotoxic activity comparable to pimozide (**Figures 1c** and **6c**), reinforcing the notion that robust CD8^+^ T cell priming does not strictly require a canonical DC maturation program. Notably, this profile contrasts with classical TLR stimuli, which simultaneously promote cross-presentation alongside robust dendritic cell maturation (53, 77, 78). In consequence, pharmacological modulation of intracellular antigen-processing pathways can enhance cross-presentation while maintaining a restrained inflammatory activation state.

An important observation of our findings is that pimozide enhanced cross-presentation not only in cDC1 but also in cDC2, suggesting that its mechanism does not simply amplify an intrinsic cDC1-restricted program but rather targets conserved intracellular processes that regulate antigen fate following endocytosis. By functionally elevating the cross-presenting competence of both populations, pimozide broadens the pool of DC subsets capable of priming cytotoxic T lymphocytes, supporting the concept that intracellular antigen-handling pathways can be leveraged to expand the contribution of multiple dendritic cell subsets to CD8^+^ T cell responses. Since cDC1 and cDC2 provide complementary, non-redundant functions to shape antitumor immunity, strategies incorporating multiple DC subsets through functional reprogramming have been associated with improved clinical outcomes (21–23).

Mechanistically, our data are consistent with pimozide as a regulator of early endolysosomal events that determine antigen fate following internalization. Cross-presentation can proceed through two principal routes: a vacuolar pathway in which peptides are generated within endocytic compartments, and a cytosolic pathway that requires antigen translocation into the cytosol followed by proteasomal degradation and TAP-dependent peptide loading (4). Our results strongly support the involvement of the cytosolic pathway, as proteasome inhibition abrogated the enhancement mediated by pimozide and β-lactamase translocation assays demonstrated increased antigen access to the cytosol. These findings indicate that pimozide enhances endolysosomal escape, one of the key steps of the cytosolic route (6, 55). Several studies in the field have shown that maintenance of a mildly acidic and non-degradative endosomal environment, largely controlled by NADPH oxidase–dependent ROS production, preserves antigen integrity while facilitating controlled membrane destabilization required for antigen export (8, 9, 56, 57, 62). In agreement with this paradigm, we observed that pimozide reduced endosomal acidification and attenuated proteolytic degradation -particularly in cDC1- without affecting antigen uptake, indicating that the drug selectively modulates post-endocytic processing rather than antigen internalization itself. Interestingly, pimozide does not significantly change antigen degradation in cDC2 despite enhanced cross-presentation in both subsets. cDC1 are characterized by relatively mild endosomal acidification and tightly regulated proteolytic activity, conditions that are already optimized to preserve antigenic substrates for cross-presentation (6, 8). In this context, modest alterations in endosomal maturation induced by pimozide may be sufficient to further limit proteolysis and shift antigen fate toward preservation. In contrast, cDC2 typically exhibit more acidic and proteolytically active endosomal compartments (8), which may confer a higher buffering capacity against changes in pH or protease activity.

In line with its effects on endosomal processing, pimozide induced robust intracellular ROS production, and pharmacological ROS scavenging abrogated the enhancement of cross-presentation. Physiological ROS production in dendritic cells has been shown to exert pleiotropic effects on endosomal compartments, including proton consumption–mediated alkalinization, attenuation of proteolytic activity, and promotion of lipid peroxidation–driven membrane destabilization (6, 8–11, 56, 62). Rather than representing independent phenomena, these processes are increasingly understood as coordinated outputs of NOX2 activity that collectively shape antigen fate within endocytic compartments. In this context, the recruitment of Galectin-3 to endosomal membranes in pimozide-treated DCs provides direct evidence of membrane damage and confirm that ROS-driven permeabilization constitutes a functional step in antigen cross-presentation. Importantly, this type of membrane disruption is thought to be partial and tightly regulated, allowing the release of internalized antigens into the cytosol without causing widespread organelle rupture or compromising cellular viability — a form of controlled permeabilization that has emerged as a key mechanism enabling antigen export while preserving compartmental integrity (6, 9, 56, 57). These observations reinforce the concept that endolysosomal membrane destabilization is not merely a byproduct of oxidative stress but a physiologically exploited process in dendritic cells. In this framework, pimozide amplifies an intrinsic redox-dependent program that coordinates endosomal alkalinization, restrained proteolysis and increased membrane permeability, thereby optimizing the efficiency of antigen escape and subsequent cytosolic processing.

Endosomal membrane destabilization can, in certain contexts, trigger inflammasome activation, particularly through the NLRP3 sensor (79, 80). However, our experiments with NLRP3-deficient dendritic cells demonstrate that pimozide-mediated enhancement of cross-presentation occurs independently of this pathway. These findings indicate that, although pimozide promotes endosomal membrane perturbation, this process does not reach the threshold required to engage inflammasome signaling, consistent with the absence of IL-1β secretion in pimozide-treated DCs. This distinction is particularly relevant, as it indicates not all forms of endolysosomal damage are functionally equivalent. While extensive membrane rupture is typically associated with the release of danger signals and activation of inflammatory pathways, more limited, spatially restricted permeabilization can support antigen translocation without triggering innate immune sensors. In this context, the Galectin-3 recruitment observed in pimozide-treated cells is consistent with a model of controlled membrane damage that is sufficient to enable antigen escape while preserving overall compartmental integrity. Together, these observations support the existence of a functional window in which endosomal membrane destabilization promotes cross-presentation without eliciting inflammasome activation. Within this window, pimozide fine-tunes membrane permeability to enhance antigen export while avoiding excessive inflammatory signaling.

While several studies have characterized pimozide as a potent STAT5 inhibitor in various tumor cells (31, 37, 38, 40), we show by Western blot that pimozide treatment of DCs reduces both total and phosphorylated STAT5, extending this activity to dendritic cells and connecting it, to our knowledge for the first time, to the regulation of antigen cross-presentation. Notably, pharmacological inhibition of STAT5 with STAT5-IN-1, a structurally unrelated inhibitor, replicated the enhancement of cross-presentation and CD8^+^ T cell activation observed with pimozide, indicating that STAT5 may function as a previously unrecognized regulator of dendritic cell cross-presenting capacity. Although STAT5 is well known for its role in cytokine signaling and lymphocyte biology, its contribution to dendritic cell antigen processing has, to our knowledge, remained largely unexplored. Importantly, STAT5-IN-1 enhanced cross-presentation without increasing ROS production. This finding argues against a simple linear model in which inhibition of STAT5 is responsible for the pimozide-induced increase in ROS and suggests that STAT5 modulation and ROS-dependent endosomal remodeling may represent mechanistically distinguishable regulatory components that converge on enhanced cross-presentation, consistent with a broader reprogramming of dendritic cell physiology rather than the engagement of a single linear pathway. A limitation of the present study is that STAT5 involvement was assessed pharmacologically: although pimozide reduces STAT5 protein content in DCs and an independent STAT5 inhibitor replicates the phenotype, causality has not been established through genetic loss- or gain-of-function approaches. DC-specific conditional *Stat5* deletion, CRISPR-mediated knockout, or STAT5 re-expression in pimozide-treated DCs represents a priority for future studies aimed at dissecting the individual contributions of each mechanistic node identified here.

The intracellular reprogramming induced by pimozide translated into enhanced functional immunity *in vivo*. Immunization with pimozide-conditioned, antigen-loaded dendritic cells resulted in robust expansion of antigen-specific CD8^+^ T cells, accompanied by increased effector cytokine production, enhanced degranulation and, superior cytolytic activity. These findings demonstrate that targeted pharmacological modulation of antigen-processing pathways is a powerful tool to enhance cytotoxic T lymphocyte priming and effector function *in vivo*. From a translational perspective, these results highlight the therapeutic potential of selectively enhancing cross-presentation within dendritic cells as a strategy to optimize CD8^+^ T cell immunity. Notably, the approach used here — ex vivo conditioning and antigen loading of dendritic cells prior to immunization — avoids systemic drug exposure altogether, and is conceptually aligned with DC-based vaccination platforms already evaluated in the clinic. This distinction is relevant because the established pharmacology of pimozide, including its narrow approved dosing range, its potential for QT-interval prolongation and its CYP2D6/CYP3A4-dependent metabolism in its current clinical indication (Tourette syndrome) (81–83), is likely to constrain its direct systemic use as an immunotherapeutic agent. Extending these findings beyond the ex vivo setting would therefore require approaches that confine pimozide activity to dendritic cells, such as antibody-conjugated formulations or DC-targeted nanoparticle carriers, which warrant further exploration in future work. Collectively, our findings establish pimozide as a pharmacological enhancer of dendritic cell cross-presenting function and provide a conceptual framework for the repositioning of small molecules as DC-targeted immunomodulators. Future studies should address whether pimozide-mediated reprogramming extends to human dendritic cell subsets and whether its combined use with checkpoint inhibitors or antigen-loading strategies could further amplify cytotoxic immunity in clinically relevant models. More broadly, the identification of STAT5 and endolysosomal physiology as pharmacologically actionable nodes in dendritic cell biology opens new avenues for the rational design of next-generation immunotherapies aimed at harnessing CD8^+^ T cell responses in cancer and infectious disease.

## Data Availability

The raw data supporting the conclusions of this article will be made available by the authors, without undue reservation.

## Glossary

AF647: Alexa Fluor 647
AIGC: Artificial Intelligence Generated Content
ANOVA: Analysis of variance
APC: Antigen-presenting cell
ATP: Adenosine triphosphate
BMDCs: Bone marrow-derived dendritic cells
CCF4: Cephalosporin-based fluorescent substrate
cDC: Conventional dendritic cell
cDC1: Type 1 conventional dendritic cell
cDC2: Type 2 conventional dendritic cell
CpG-ODN: CpG oligodeoxynucleotide
CPRG: Chlorophenol red-β-D-galactopyranoside
CQ: Chloroquine
CTL: Cytotoxic T lymphocyte
DC: Dendritic cell
DMSO: Dimethyl sulfoxide
DPI: Diphenyleneiodonium
DQ-OVA: DQ-ovalbumin (self-quenched fluorescent substrate)
eF670: eFluor 670
ELISA: Enzyme-linked immunosorbent assay
ER: Endoplasmic reticulum
ERAD: ER-associated degradation
FBS: Fetal bovine serum
Flt3L: FMS-like tyrosine kinase 3 ligand
GM-CSF: Granulocyte-macrophage colony-stimulating factor
H2-DCFDA: 2′, 7′-dichlorodihydrofluorescein diacetate
H_2_O_2_: Hydrogen peroxide
IFN-γ: Interferon gamma
IFN-β: Interferon beta
IL-1α: Interleukin-1 alpha
IL-1β: Interleukin-1 beta
IL-6: Interleukin-6
IL-10: Interleukin-10
IL-12p70: Interleukin-12 p70
IL-17A: Interleukin-17A
IL-23: Interleukin-23
IL-27: Interleukin-27
LLOMe: L-leucyl-L-leucine methyl ester
LPS: Lipopolysaccharide
MCP-1: Monocyte chemoattractant protein-1
MEMα: Minimum Essential Medium alpha
MFI: Mean fluorescence intensity
MHC I: Major histocompatibility complex class I
MHC II: Major histocompatibility complex class II
NAC: N-acetylcysteine
NIH: National Institutes of Health
NLRP3: NOD-like receptor protein 3
NOX2: NADPH oxidase 2
NADPH: Nicotinamide adenine dinucleotide phosphate
OVA: Ovalbumin
OVA-AF647: Alexa Fluor 647-conjugated ovalbumin
p-STAT5: Phosphorylated STAT5
PBS: Phosphate-buffered saline
pDC: Plasmacytoid dendritic cell
pHrodo: pH-sensitive fluorescent dye
Poly(I:C): Polyinosinic-polycytidylic acid
PolyU: Polyuridylic acid
ps: Polystyrene
PVDF: Polyvinylidene fluoride
ROS: Reactive oxygen species
RT: Room temperature
S8: SIINFEKL peptide (OVA_257-264_)
SDS-PAGE: Sodium dodecyl sulfate polyacrylamide gel electrophoresis
SEM: Standard error of the mean
SIINFEKL: OVA-derived peptide (single-letter amino acid code) presented by H-2K^b^
STAT5: Signal transducer and activator of transcription 5
TAP: Transporter associated with antigen processing
TCR: T cell receptor
TLR: Toll-like receptor
TNF-α: Tumor necrosis factor alpha
WT: Wild-type
