# Supplementary material for: Pimozide enhances antigen cross-presentation and promotes cytotoxic CD8+ T cell immunity by stimulating dendritic cells
Source: Front Immunol. 2026 Oct 1;17:1946712. doi: 10.3389/fimmu.2026.1946712 (PMC13668838; doi:10.3389/fimmu.2026.1946712)
Supplement: Supplementary file 1 [file DataSheet1.docx]

**Pimozide enhances antigen cross-presentation and promotes cytotoxic CD8⁺ T cell immunity by stimulating dendritic cells.**

**Luz María Palacios^1,2^, Nicolás Daniel Dho^1,2^, Cintia Araujo Furlán^1,2^, Guadalupe López Menichetti^1,2^, Federico Ruiz Moreno^1,2^, Laura Guantay^1,2^, Sofia Daiana Castell^1,2^, María Esperanza Felici^1,2^, Gastón Soria^1,2#^, Belkys Maletto^1,2^, María Inés Crespo^1,2*^, Gabriel Morón^1,2*^.**

^1^Universidad Nacional de Córdoba, Facultad de Ciencias Químicas, Departamento de Bioquímica Clínica. Córdoba, Argentina.

^2^Consejo Nacional de Investigaciones Científicas y Técnicas (CONICET), Centro de Investigaciones en Bioquímica Clínica e Inmunología (CIBICI). Córdoba, Argentina.

#Present address: OncoPrecision Corporation. New York, NY, United States.

*Corresponding author: María Inés Crespo (ines.crespo@unc.edu.ar) and Gabriel Morón (gmoron@unc.edu.ar)

**SUPPLEMENTARY MATERIAL**

**LEGENDS OF SUPPLEMENTARY FIGURES**

**Supplementary Figure 1**

**Pimozide does not alter dendritic cell viability or cytokine secretion profile**

**a)** Representative MTT viability assay data from DCs treated with 15 µM pimozide (P) or 5 µg/ml CpG-ODN for 3 h at 37°C. **b)** Gating strategy used to analyze CD8^+^ T cell proliferation assays. **c)** Gating strategy to identify cDC1 and cDC2 subsets from Flt3L BMDCs cultures. **d)** Cytokine production by DCs treated with pimozide or CpG-ODN. DCs were incubated with 15 µM P or 5 µg/mL CpG-ODN for 12 h at 37°C. Cell culture supernatants were collected, and cytokine production was measured using Legendplex™. Heat map shows cytokine concentration, expressed in pg/mL. Statistical analysis was conducted using one-way ANOVA with Tukey’s multiple comparisons test. Data are presented as mean ± SEM of at least two independent experiments (n≥3). Significance levels: p<0.01 (**), and p<0.0001 (****). Non-significant differences (p>0.05) are indicated with their corresponding p values.

**Supplementary Figure 2**

**a) OVA cross presentation by JAWS II cells:** JAWS II cells were incubated with 3.25, 7.5 or 15 µM pimozide (P) and 1 mg/mL OVA for 5 h at 37°C. Then, cells were extensively washed and co-cultured with B3Z hybridoma cells for 18 h. B3Z activation was assessed using a colorimetric assay. Data are shown as fold change of absorbance relative to the condition without P. **b) Positive and negative control of antigen translocation:** Representative controls for the antigen translocation assay. JAWS II cells were pulsed with β-lactamase at 37°C and treated with PR-169 or LLOMe for 2 h. Cells incubated with CCF4-AM in the absence of β-lactamase served as a negative control for substrate cleavage (first panel). Baseline translocation was measured in β-lactamase–pulsed cells without additional stimulation (second panel). PR-619 (third panel) and LLOMe (fourth panel) were used as a negative and positive control for translocation, respectively. Statistical analysis was conducted using two-way ANOVA with Tukey’s multiple comparisons test. Data are presented as mean ± SEM of at least two independent experiments (n≥3). Significance levels: p<0.01 (**), and p<0.001 (***). Non-significant differences (p>0.05) are indicated with their corresponding p values.

**Supplementary Figure 3**

**Pimozide enhances cross-presentation in dendritic cells independently of the NLRP3 inflammasome.**

**a)** DCs from C57BL/6 wild-type (WT) or NLRP3-deficient (NLRP3 KO) mice were incubated with 0.25 mg/mL OVA in the presence or absence of 15 µM P for 1.5 h at 37°C. Treated DCs were then co-cultured with eF670-labeled CD8^+^ T cells isolated from OT-I mice. After 72 h, cells were harvested, stained for surface markers, and culture supernatants were collected for cytokine analysis. Left: representative dot plot analysis showing the expression of CD44 vs eF607 staining. Right: Quantification of frequency of eF670^low^ CD44^+^ CD8^+^ T cells and IFN-γ concentration in culture supernatants are shown. **b)** DCs were incubated in the presence of 0.1 ug/mL LPS (from E. coli, strain 0111:B4), 4 mM ATP or with 0.1 ug/mL LPS for 3h alone plus 4 mM ATP for the rest of the incubation time. After 18 h, supernatants were collected and IL-1b was measured by sandwich ELISA. **c)** DCs were incubated with 0.25 µg/mL Alexa Fluor 647–conjugated OVA (OVA-AF647) in the presence of 15 µM pimozide for 30, 60, and 90 min at 37°C. Antigen uptake was quantified as the Mean Fluorescence Intensity of AF647 in OVA-AF647^+^CD11c^+^MHCII^+^ cells by flow cytometry. **d)** DCs were incubated with 15 µM P or 0.5 mM LLOMe for 2 h at 37°C. Cells were fixed, permeabilized and stained with PE anti-Galectin-3 and Hoechst. Galectin-3 (Gal-3) staining was quantified as the ratio of total Gal-3 fluorescence intensity (FI) to cytosolic FI per cell (left). Representative images acquired at 60X magnification are shown (right).

Statistical analysis was conducted using two-way (**a**, **b** and **c**) or one-way (**d**) ANOVA and Tukey’s multiple comparisons test. Data are presented as mean ± SEM of at least two independent experiments (n≥3). Significance levels are denoted as p<0.05 (*) and p<0.001 (***). Non-significant differences (p>0.05) are indicated with their corresponding p values.

**Supplementary Figure 4**

**STAT5 modulation by pimozide in DCs**

**a)** Representative uncropped Western blot images corresponding to STAT5 and p-STAT5 detection in DCs treated with pimozide (P) or GM-CSF. **b)** Quantification of STAT5 (left panel) and phospho-STAT5 levels (right panel) in GM-CSF treated DCs, expressed as fold change relative to OVA-treated DCs. Data are presented as mean ± SEM of at least two independent experiments (n≥3). **c)** ROS quantification in DCs incubated with STAT5 inhibitor. DCs were incubated with 15 μM STAT-IN-1 or DMSO for 2 h. Then, cells were culture in the presence of 1 µM H2-DCFDA probe for 30 min at 37°C. Quantification of ROS production in CD11c^+^ MHCII^+^ cells was performed by flow cytometry and expressed as fold change in H2-DCFDA MFI relative to DMSO-treated controls. Statistical analysis was conducted using t-test. Significance levels are denoted as p<0.05 (*), and p<0.001 (***). Non-significant differences (p>0.05) are indicated with their corresponding p values.

**Supplementary Figure 5**

**Experimental workflow and gating strategies for the analysis of antigen-specific CD8⁺ T cell responses in vivo**

**a)** Schematic representation of the immunization protocol and experimental timeline used for in vivo studies. **b)** Gating strategy used to identify CD107a^+^ and IFN-γ^+^ CD8⁺ T cells. **c)** Gating strategy used to identify OVA-specific CD8⁺ T cells. **d)** Gating strategy used to assess the in vivo cytotoxic T lymphocyte (CTL) assay.

**Supplementary Table 1. Antibodies and other reagents used for cell labeling in fluorescence-based techniques.**

| **Specificity** | **Clon** | **Isotype** | **Fluorochrome** | **Company** |
| --- | --- | --- | --- | --- |
| **CD11b** | M1/70 | R IgG2b, κ | eF450 | Biolegend |
| **CD11b** | M1/70 | R IgG2b, κ | eF660 | Biolegend |
| **CD11b** | M1/70 | R IgG2b, κ | FITC | Pharmigen |
| **CD11c** | N418 | H IgG | PE-Cy7 | Biolegend |
| **CD11c** | N418 | H IgG | BV421 | Biolegend |
| **CD11c** | N418 | H IgG | APC- EF780 | eBioscience |
| **CD24** | M1/69 | R IgG2b, κ | PE-Cy5 | Invitrogen |
| **SIRPa** | P84 | R IgG1, κ | FITC | Biolegend |
| **CD3ε** | 17A2 | R IgG2b, κ | FITC | Biolegend |
| **CD3ε** | 145-2C11 | H IgG | BV 711 | Biolegend |
| **CD44** | IM7 | R IgG2b, κ | PE-Cy5 | eBioscience |
| **CD80** | 16-10A1 | H IgG2, κ | FITC | Pharmigen |
| **CD86** | GL1 | R IgG2a, κ | PE | eBioscience |
| **CD8α** | 53-6.7 | R IgG2a, κ | PE-Cy7 | Biolegend |
| **CD8α** | 53-6.7 | R IgG2a, κ | FITC | Pharmigen |
| **IFN-g** | XMG1.2 | R IgG1, κ | FITC | IH |
| **Streptavidin** |  |  | APC | Pharmigen |
| **Streptavidin** |  |  | PE-TxRed | Invitrogen |
| **I-Ab MHC-II** | AF6-120.1 | M IgG2a, κ | SB780 | eBioscience |
| **I-Ab,d,q / I-Ed,k MHC-II** | M5/114.15.2 | R IgG2b, κ | PE | eBioscience |
| **LAMP-1** | 1D4B | R IgG2a, κ | Biotin | Biolegend |
| **CD107a** | 1D4B | R IgG2a, κ | PE | Biolegend |
| **Galectin-3** | B2C10 | M IgG1, κ | PE | Santa Cruz Biotechnology |
| **H-2K^b^-SIINFEKL** | 25-D1.16 | M IgG1, κ | APC | eBioscience |
| **IgG mouse** | Polyclonal | Goat | PE 234V | SouthernBiotek |
| **Streptavidin** |  |  | APC | Pharmigen |
| **Streptavidin** |  |  | PE-TxRed | Invitrogen |
| **Fixable Viability Dye** |  |  | eF780 | eBioscience |
| **Fixable Viability Dye** |  |  | Ghost Violet 510 | Tonbo Bioscience |

H: hamster, R: rat, M: mouse, IH: antibodies produced in-house.

**Supplementary Table II. Capture and detection pairs of antibodies for cytokine quantification by Enzyme Linked Immunosorbent Assay.**

| **Specificity** | **Clon** | **Function** | **Company** |
| --- | --- | --- | --- |
| **IFN-**γ | R4-6A2  XMG1.2 | Capture  Detection | Biolegend |
| **IL-1β** | B122  Polyclonal sera | Capture  Detection | eBioscience |
